# Case report: Bilateral infantile acromion fractures in the setting of congenital glenoid dysplasia

**DOI:** 10.1007/s00247-026-06802-z

**Published:** 2026-10-12

**Authors:** Molly Crosswell, Kelley Marshall Johnson, Amanda Small, Kenneth W. Martin, Mary-Fran R. Crosswell

**Affiliations:** 1 University of South Carolina School of Medicine Greenville, 607 Grove Rd, Greenville, SC 29605 USA; 2 Children’s Healthcare of Atlanta, Atlanta, GA USA; 3 Prisma Health, Greenville, SC USA; 4 St. Christopher’s Hospital for Children, Philadelphia, PA USA

**Keywords:** acromion fractures, congenital glenoid dysplasia, glenoid deformity

## Abstract

We report a rare case of a 5-week-old male who presented with isolated acromion fractures occurring bilaterally 10 days apart. As scapular fractures are a high-specificity fracture for abusive injury, these findings were concerning for child abuse. The patient was later diagnosed with congenital glenoid dysplasia. Given this diagnosis with an otherwise normal medical evaluation for physical abuse, this case was deemed indeterminate for abusive injury. The multidisciplinary team consensus was that the injuries were likely fractures resulting from restriction of normal range of glenohumeral motion caused by his glenoid deformity. Literature on congenital glenoid dysplasia is limited in the pediatric population. This case highlights the need for further investigation into early presentations of congenital glenoid dysplasia and its association with acromion fractures.

## Introduction

Congenital glenoid dysplasia is a primary abnormality involving the abnormal formation and subsequent ossification of the bony glenoid and adjacent scapular neck, which often occurs bilaterally [1]. Congenital glenoid dysplasia should be distinguished from glenohumeral dysplasia, which is a secondary, acquired deformity of the glenohumeral joint and most often a sequela of brachial plexus birth palsy, in which muscle imbalance produces progressive glenoid retroversion and posterior subluxation of the humeral head, typically presenting unilaterally [2]. Congenital glenoid dysplasia is likely underdiagnosed and may present with pain, instability, stiffness, and decreased range of motion. However, it is often asymptomatic, particularly in the pediatric population, making early diagnosis rare [3]. As presenting symptoms can vary, radiographic recognition is essential for diagnosis. Although congenital glenoid dysplasia has been associated with shoulder instability and altered biomechanics, to our knowledge, there has not been a previously reported association with acromion fractures. Scapular fractures, including acromion fractures, are rare in the pediatric population and are considered highly concerning for child abuse when present in the absence of high-energy trauma [4]. In suspected cases of physical abuse, scapular fractures can present bilaterally and are often associated with other traumatic findings, such as additional fractures or CNS injury [5]. Given their diagnostic significance, scapular fractures should be carefully evaluated.

This case highlights a rare association between congenital glenoid dysplasia and bilateral acromion fractures in infancy and underscores the importance of considering congenital disorders when evaluating fractures for physical abuse.

### Case presentation

**Fig. 1:**
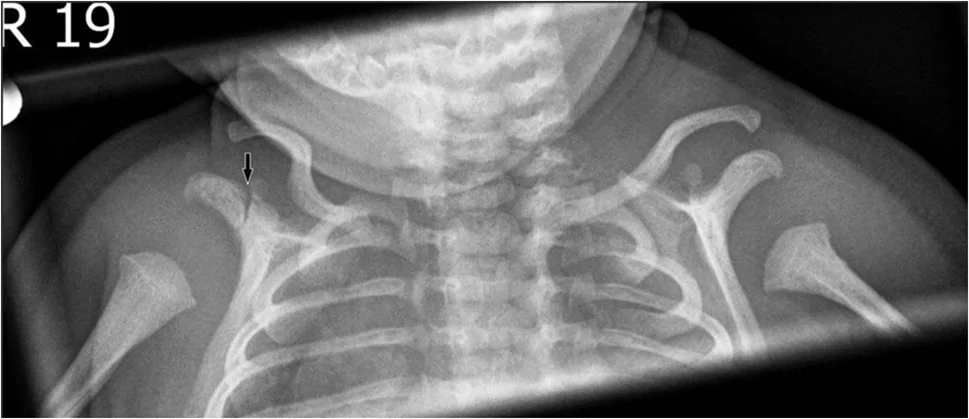
Anteroposterior (AP) bilateral clavicle radiograph, a 5-week-old male, demonstrates unilateral right-sided scapular fracture through the basi-acromion without evidence of healing (*arrow*). The basi-acromion is defined as the lateral extension of the scapular spine which forms the root of the acromion representing the ossified portion of the acromion at birth [6]

A 5-week-old fraternal male twin presented to the local pediatric emergency department with decreased right upper extremity movement for 24 h. The patient was otherwise healthy with no pre-existing upper extremity pain or weakness, the product of an unremarkable cesarean birth, and he presented with no history or visible signs of trauma. On examination, the patient had questionable tenderness to palpation of his right shoulder. Radiograph of bilateral clavicles revealed a minimally displaced right basi-acromion fracture (Fig. 1). He was admitted to the hospital and the Child Abuse Pediatrics team was consulted. His workup included a normal non-sedated MRI of the brain and cervical spine, a normal screen for occult abdominal trauma (including liver transaminases and pancreatic enzymes), and a skeletal survey which identified only the known right acromion fracture. His fraternal twin had a normal medical evaluation, and her skeletal survey was negative for fractures. A report to child protective services was made, and the patient was discharged home to parents with supervision by a relative in the home.

**Fig. 2:**
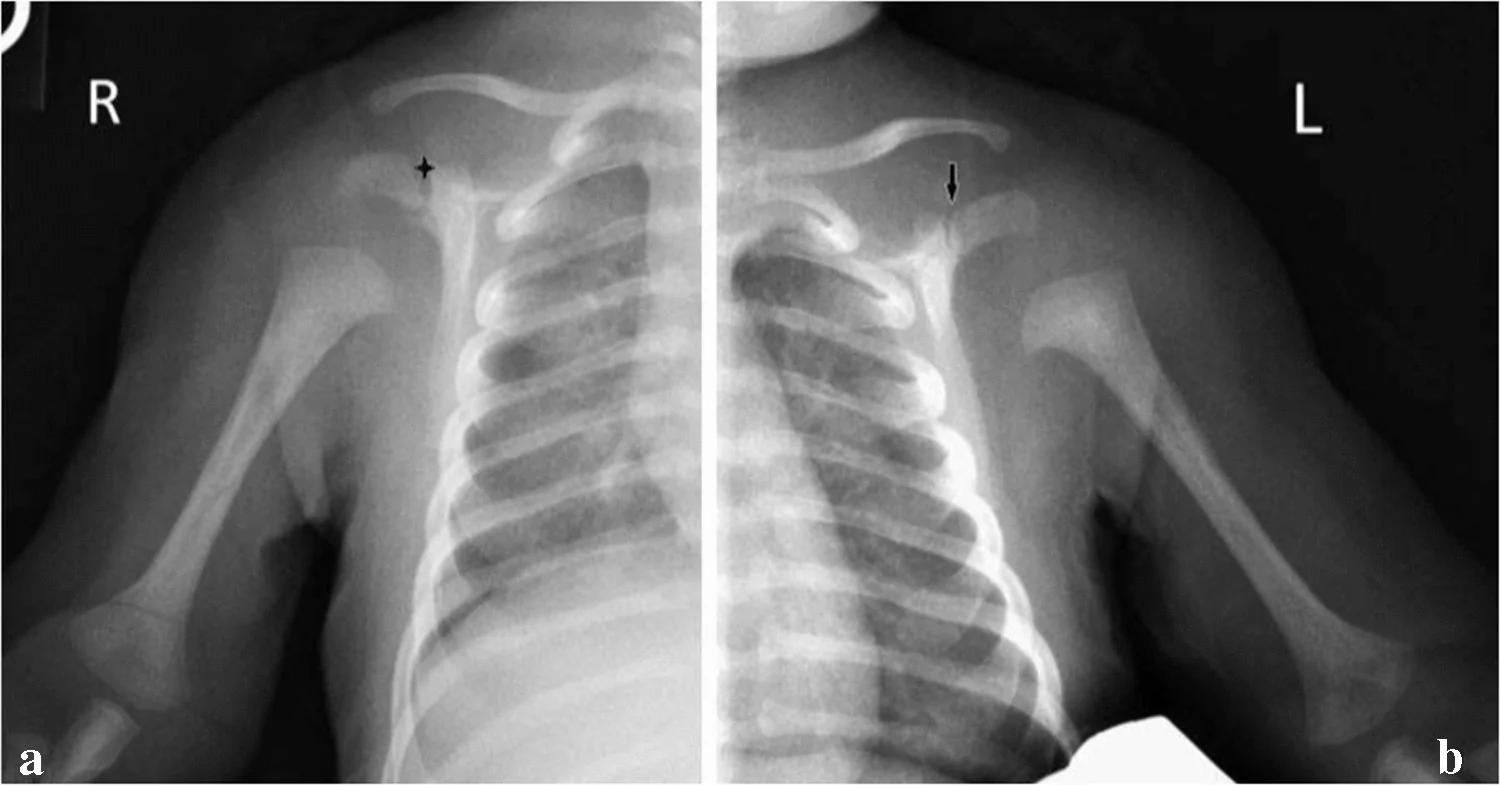
AP right humerus radiograph **a**, and AP left humerus radiograph **b**, obtained 10 days later, now a 6-week-old male, demonstrates new left-sided acromial fracture (*arrow*). The subacute right acromial fracture demonstrates peri-fracture resorptive changes with increased fracture displacement (*star*)

**Fig. 3:**
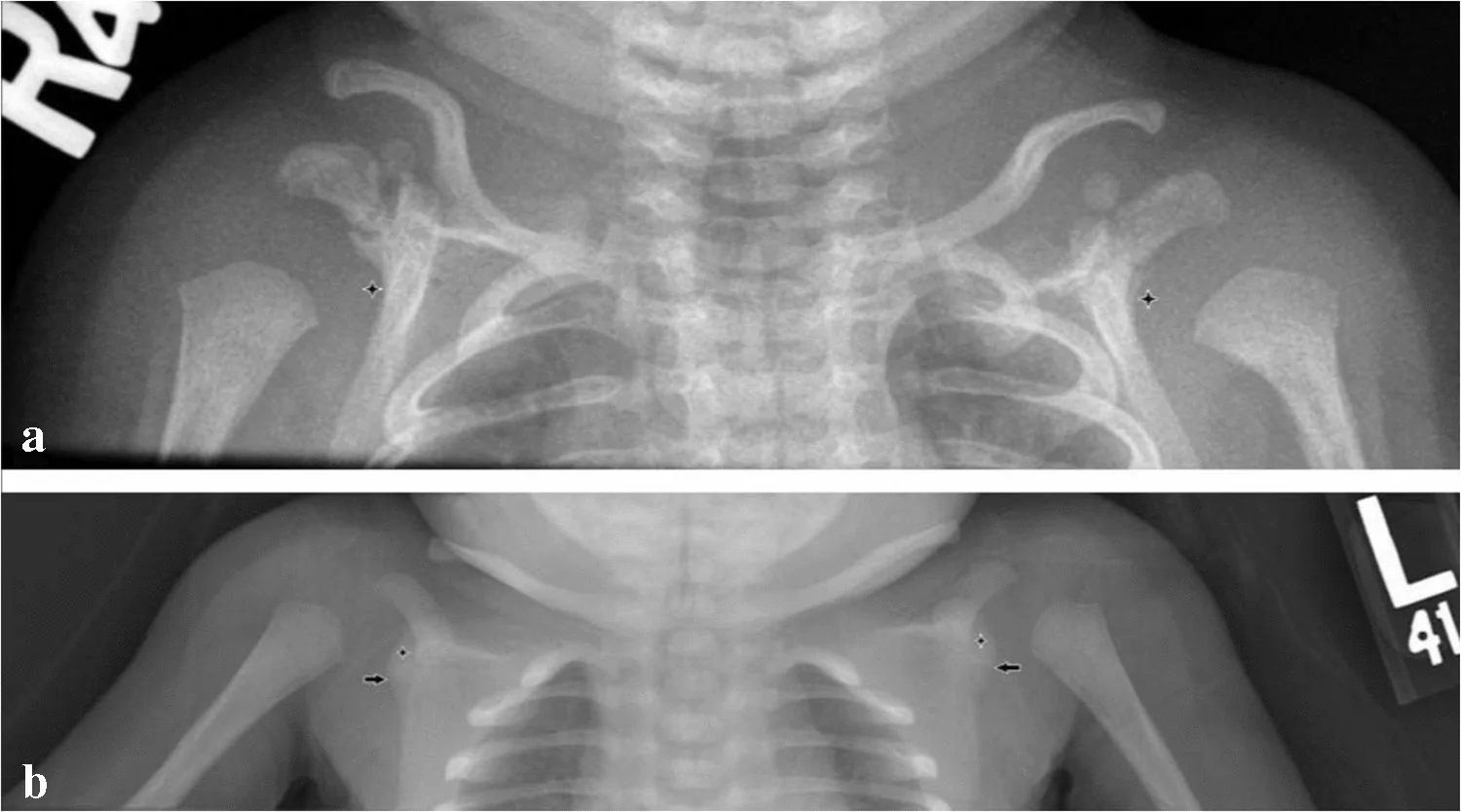
AP bilateral clavicle radiograph, case study, now a 7-week-old male **a**. Note the absence of the normal glenoid face anatomy with a pronounced concavity in the region of the scapular neck (*star*). AP bilateral clavicle radiograph newborn comparison **b** reveals the normal central glenoid cup (*star*) with superior tilt and a well-defined lateral promontory (*arrow*) at the posterior inferior glenoid margin. Normally, the glenoid faces anterolaterally and superiorly, forming an angular bony prominence or “inferior glenoid promontory” of 40–50° at the junction of the glenoid with the lateral border of the scapula [6]

**Fig. 4:**
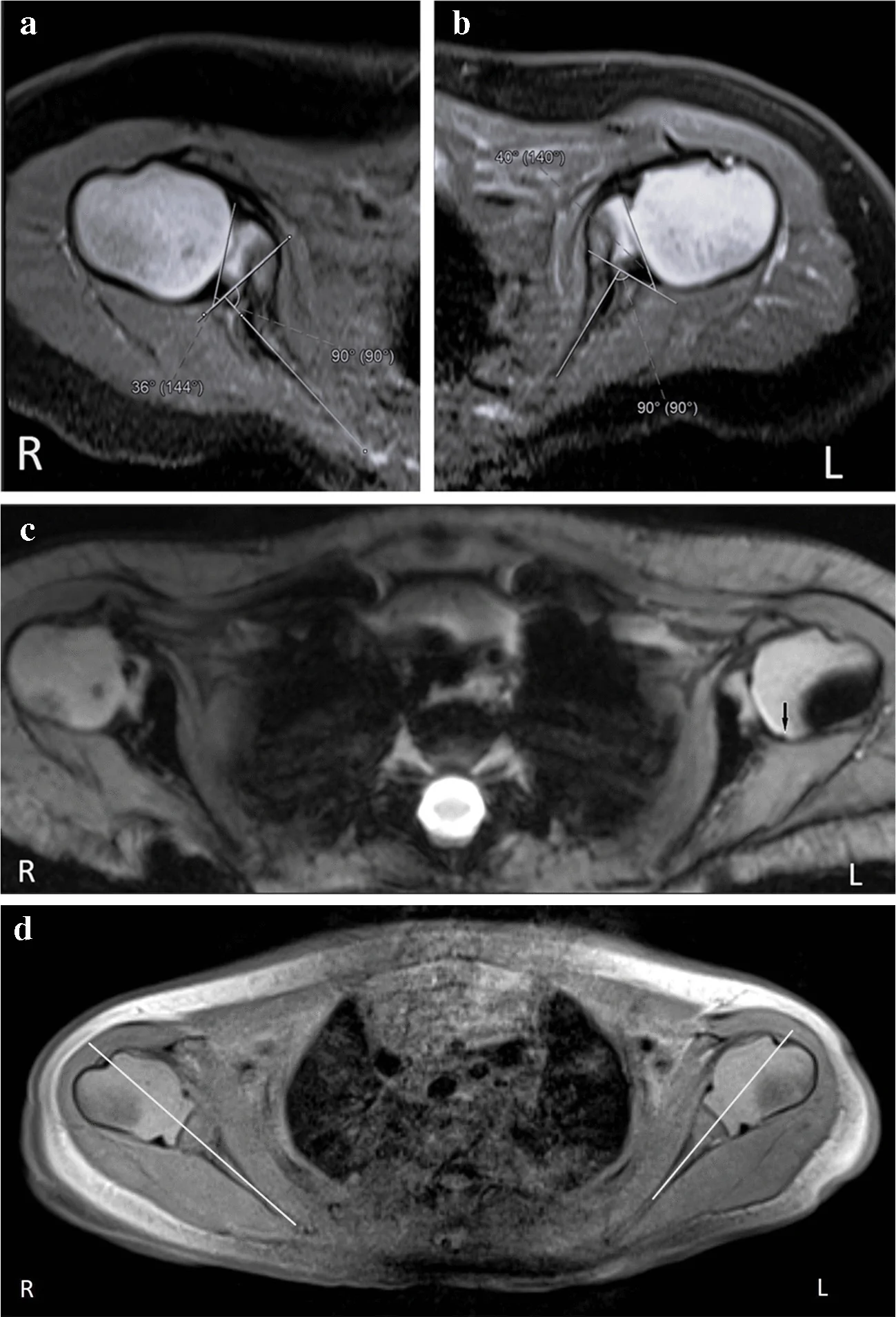
Case study bilateral shoulder MRI performed at 12 weeks of age **a**-**c** and an age-matched control **d**. Axial proton density (PD) fat-saturated (FS) images of the right **a** and left **b** shoulders demonstrate exaggerated glenoid fossa retroversion, measuring 36° on the right and 40° on the left (normal values for age are <10° retroversion before age 2) [6]. Axial gradient recalled echo (GRE) of bilateral shoulders **c** demonstrates exaggerated retroversion of the glenoid fossa with posterior subluxation (*arrow*) of the left humeral head. Axial T1-weighted images of bilateral shoulders in a normal age-matched 3-month-old male **d**. Note the ventrally directed glenoids, the central positioning of the humeral heads within the glenoid fossae, all maintaining central alignment on the scapular spine axis (*lines*)

**Fig. 5:**
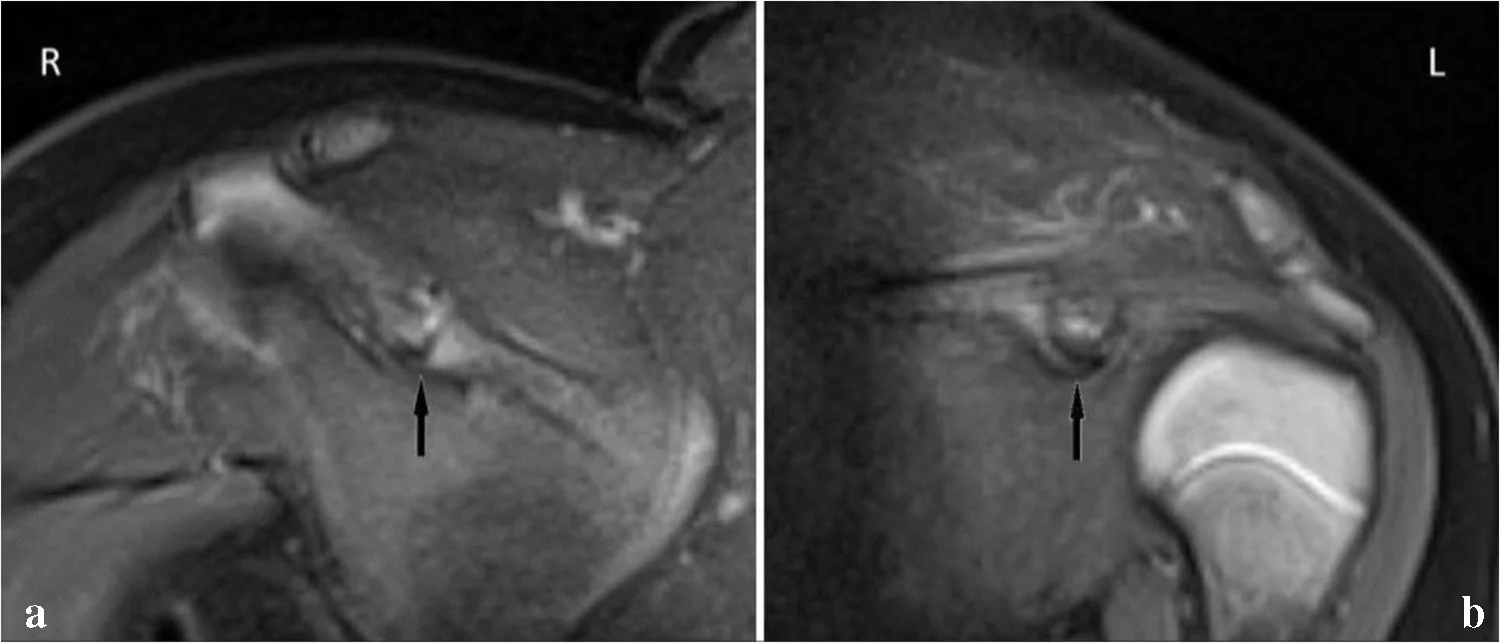
Coronal oblique FS T2WI of right **a** and left **b** scapula of a 12-week-old male case study demonstrates similarly positioned healing fractures extending through the basi-acromion bilaterally

The patient presented 10 days later to a pediatric orthopedic office with decreased movement of his left arm. Bilateral humerus radiographs demonstrated a new fracture through the left basi-acromion and the subacute acromial fracture with mild displacement on the right (Fig. 2). The patient was readmitted to the hospital, and additional work-up was obtained. A repeat MRI of the brain remained normal. A screen for metabolic bone disease included a normal free T4, thyroid-stimulating hormone, calcium, magnesium, phosphorus, alkaline phosphatase, parathyroid hormone, and 25-hydroxy-vitamin D. He was evaluated by a geneticist and whole-exome sequencing and an Ehlers-Danlos Syndrome Panel did not reveal an underlying genetic condition. A follow-up skeletal survey without skull or spinal radiographs performed 16 days after the initial skeletal survey was negative for additional fractures. Imaging was reviewed by an outside radiologist who raised the concern for glenoid hypoplasia (Fig. 3). The patient underwent bilateral shoulder MRI, interpreted by a pediatric musculoskeletal radiologist, which confirmed the diagnosis of symmetric congenital glenoid dysplasia in addition to the healing fractures occurring through the ossified basi-acromions (Figs. 4 and 5). In addition to the abnormal posterior rotation of the glenoid fossae seen on axial imaging, coronal imaging revealed exaggerated downward tilt of the patient’s glenoid fossae, with declination angles of approximately 45°, as compared to the expected normal upward glenoid tilt usually seen in childhood of 0–15° [7].

**Fig. 6:**
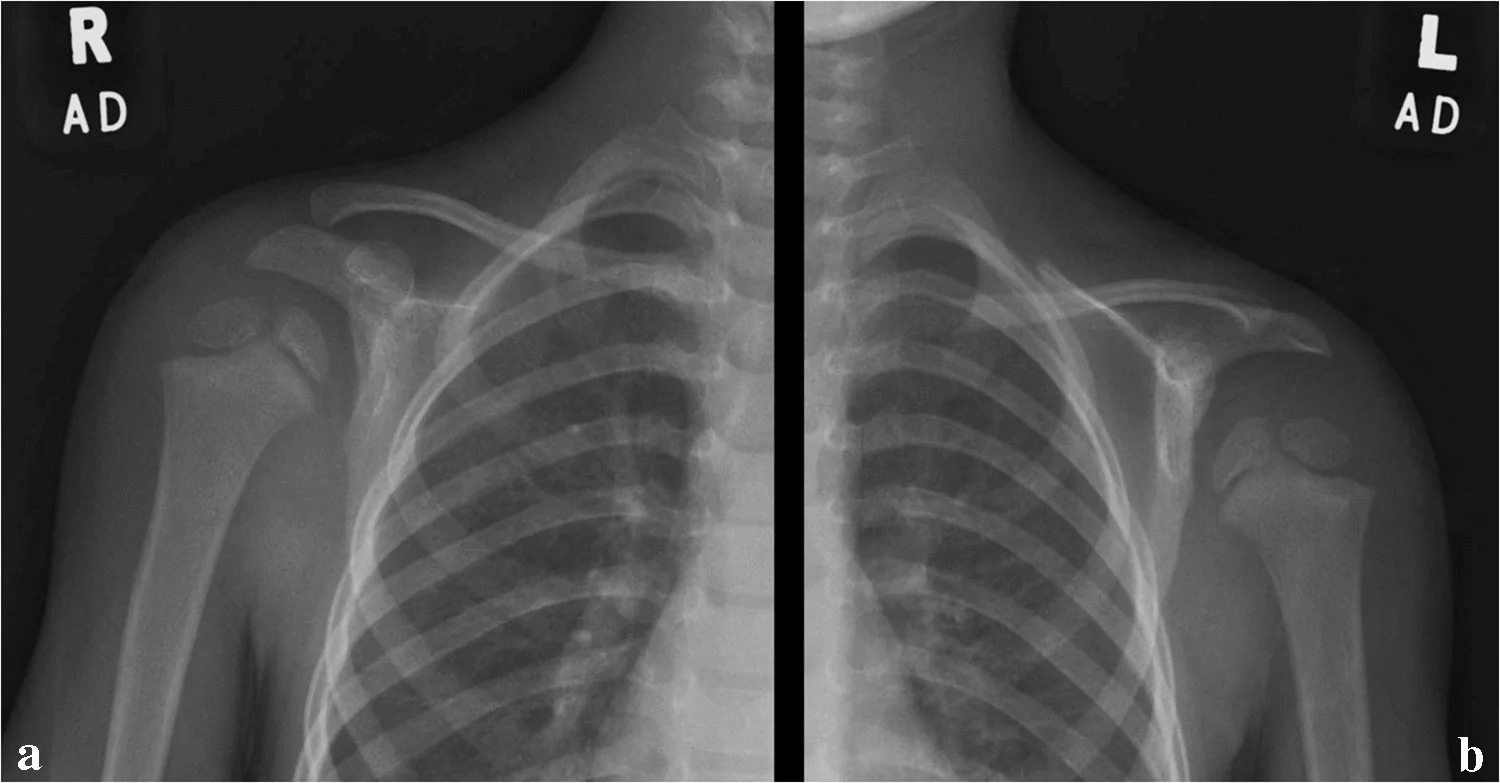
AP shoulder radiographs, case study age 2.5 years, right **a** and left **b**, highlight glenoid dysplasia morphologic changes with non-visualization of the posterior glenoid rim due to retroversion, joint laxity, with left humeral inferior subluxation, and absence of the normal promontory laterally

**Fig. 7:**
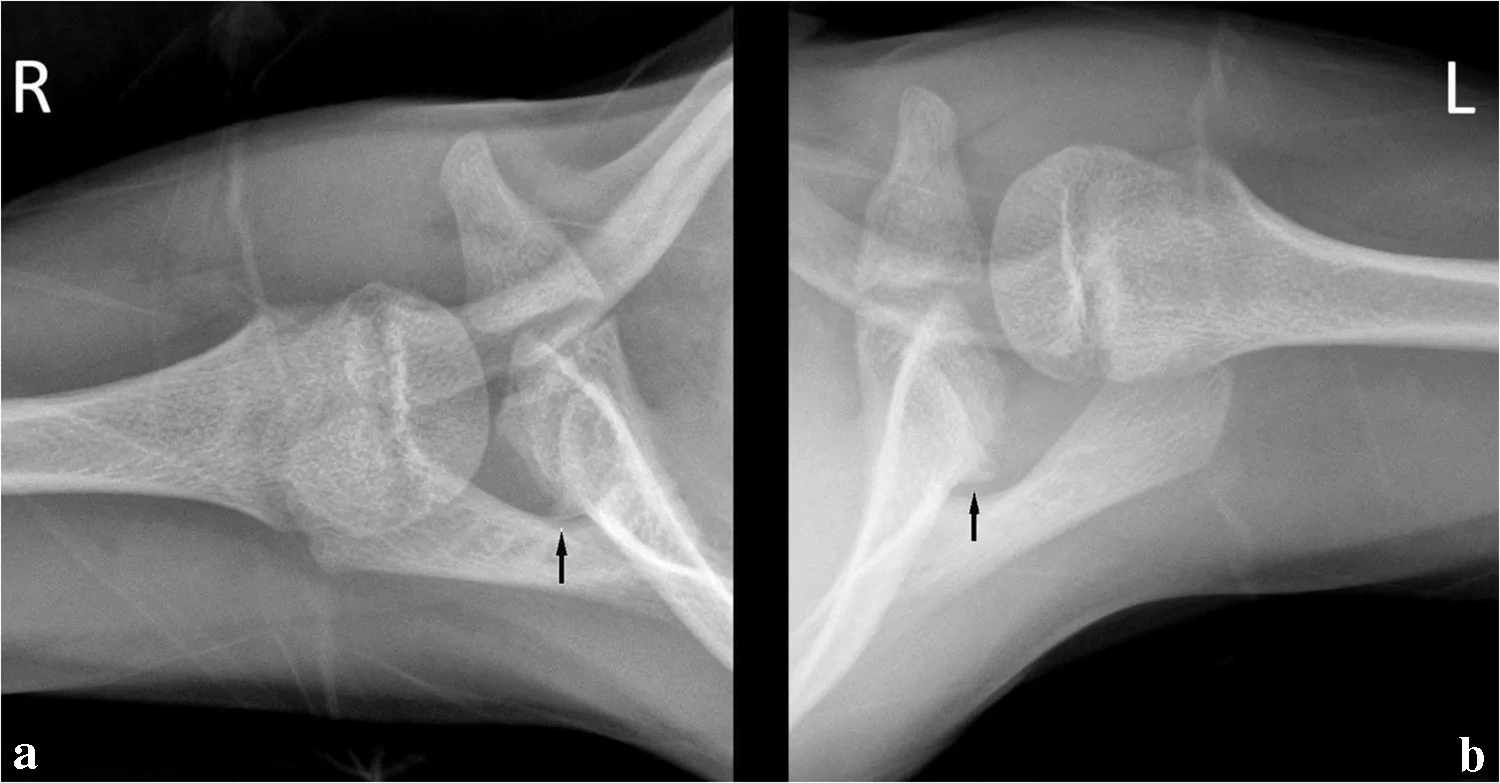
Axillary view bilateral shoulders, age 5, right **a** and left **b**, demonstrate persistent glenoid retroversion and posterior glenoid rim remodeling with scalloped margination and periosteal spurs evident (*arrows*). Right-sided posterior subluxation of the humeral head relative to scapular axis indicates persistent joint laxity

The findings were deemed indeterminate for physical abuse by the Child Abuse Pediatrics team, considering the unknown effects of altered shoulder mechanics from congenital glenoid dysplasia and the absence of any other associated injuries after a thorough and repeated evaluation. While physical abuse could not be completely ruled out, the symmetric appearance of isolated acromion fractures in the setting of congenital glenoid dysplasia made physical abuse much less likely. The bilateral acromion fractures were treated conservatively. The patient received weekly physical therapy from 6 months to 3 years of age and twice-weekly occupational therapy from 14 months to 4 years of age with goals including increasing range of motion of bilateral upper extremities, strengthening muscles in the shoulder girdle and increasing flexion/abduction shoulder movements. By age 4, he had achieved full range of motion in flexion and abduction in his bilateral upper extremities. His orthopedic surgeon released restrictions on his activity, monitoring him annually with imaging, and the patient is currently an active boy engaged in a variety of sports without subsequent issues to his shoulder girdle. Representative images obtained during yearly orthopedic surveillance confirm ongoing joint laxity and glenoid morphologic deficiencies (Figs. 6 and 7).

## Discussion

Congenital glenoid dysplasia is characterized by underdevelopment of the bony glenoid and scapular neck and may present with shoulder instability, limited range of motion, and early degenerative changes [8]. It can occur in isolation or in association with other syndromes, including Apert, Cornelia de Lange, Holt-Oram, Grant, aglossia-adactylia, and Hurler syndromes. Additionally, acquired unilateral deformities may mimic congenital glenoid dysplasia and should be ruled out [1]. Congenital glenoid dysplasia is typically diagnosed in adolescence, frequently following sports-related injuries, or in adulthood with early-onset shoulder osteoarthritis. Childhood diagnosis is rare and usually incidental [3]. Although clinical presentation varies, the condition is often bilateral and asymptomatic. Congenital glenoid dysplasia is likely underdiagnosed, with recent studies reporting an incidence of 19–35% [2]. Evaluation of congenital glenoid dysplasia can be performed with radiography, CT, or MRI. MRI is the preferred imaging technique as it allows assessment of both osseous and soft-tissue abnormalities.

This case is a unique presentation of congenital glenoid dysplasia with bilateral basi-acromion fractures presenting in infancy. To our knowledge, such an association has not been previously reported. In this case, the initial presence of an acromion fracture raised concern for abuse. The subsequent presentation of a symmetric contralateral acromion fracture suggested an underlying anatomical etiology. Cross-sectional imaging confirmed the diagnosis of congenital glenoid dysplasia, and multidisciplinary evaluation supported a non-abusive etiology. The hypothesis and consensus were that the acromion fractures developed in the setting of restricted range of shoulder motion caused by the anatomic variant. An abnormally directed glenoid, tilted downward and rotated posteriorly, would restrict the normal range of motion of the glenohumeral joint, resulting in mechanical restriction of cross-body adduction, vertical abduction, and forward flexion, motions commonly used in the care of an infant, such as in dressing and undressing.

The diagnosis of physical abuse is complex and requires evaluation of patient history, physical examination, and laboratory and radiologic findings. Underdiagnosis of physical abuse places a child at recurrent risk of harm; however, overdiagnosis carries significant consequences as well, including unnecessary removal of a child from the home and emotional trauma for all parties involved. In all cases of evaluation for physical abuse, careful radiologic review and a collaborative approach are essential to reaching the appropriate diagnosis.

This case underscores the importance of considering congenital anomalies in the differential diagnosis when evaluating infants with atypical fracture patterns. Further study into the early manifestations of congenital glenoid dysplasia and its relationship to fracture risk is warranted to better differentiate congenital findings from those associated with trauma and reduce the risk of overdiagnosis of abuse.

## Data Availability

No datasets were generated or analysed during the current study.
